# Quantitative mechanisms of DNA damage sensing and signaling

**DOI:** 10.1007/s00294-019-01007-4

**Published:** 2019-06-21

**Authors:** Susanne C. S. Bantele, Boris Pfander

**Affiliations:** Max Planck Institute of Biochemistry, DNA Replication and Genome Integrity, Martinsried, Germany

**Keywords:** DNA damage checkpoint, Signal transduction, Double strand break, DNA end resection, Cell cycle, Post-translational modification, Genome stability

## Abstract

DNA damage occurs abundantly during normal cellular proliferation. This necessitates that cellular DNA damage response and checkpoint pathways monitor the cellular DNA damage load and that DNA damage signaling is quantitative. Yet, how DNA lesions are counted and converted into a quantitative response remains poorly understood. We have recently obtained insights into this question investigating DNA damage signaling elicited by single-stranded DNA (ssDNA). Intriguingly, our findings suggest that local and global DNA damage signaling react differentially to increasing amounts of DNA damage. In this mini-review, we will discuss these findings and put them into perspective of current knowledge on the DNA damage response.

In order to ensure genome stability, cells need to detect DNA lesions such as DNA double-stranded breaks (DSBs) and signal their presence so that an appropriate cellular response is triggered (Zhou and Elledge 2000; Harrison and Haber 2006; Ciccia and Elledge 2010). The central importance of the networks carrying out this fundamental task is underscored by the observation that mutations in DNA damage signaling proteins often coincide with a predisposition for cancer development and progeria (O’Driscoll 2012).

DNA damage signaling is commonly understood to be a quantitative process, which generates a response appropriate to the cellular damage load (Zierhut and Diffley 2008; Balogun et al. 2013; Clerici et al. 2014; Ira et al. 2004; Mantiero et al. 2007). It is, however, unclear how such a quantitative response is generated in molecular terms and how the dynamic range of the response is tuned.

DNA damage signaling is mediated by proteins of the DNA damage checkpoint, which can recognize DNA structures that indicate the presence of DNA lesions and convert them into downstream DNA damage signals. Single-stranded DNA (ssDNA) marks sites of DNA damage and can be considered an upstream DNA damage signal that is recognized by proteins of the DNA damage checkpoint in order to transduce this upstream signal into a downstream checkpoint signal. While ssDNA is inherently generated during DNA replication and transcription, the presence of DNA lesions often triggers extensive ssDNA formation (Zou 2007). For example, at DSBs ssDNA is generated by a process called DNA end resection (Sugawara and Haber 1992; Symington 2014).

What information does the amount of ssDNA in a cell hold? First, it gives information about the number of lesion sites, as many lesions will obviously expose more ssDNA than few. Second, it could potentially tell us about the persistence time of a given DNA lesion, at least in the case of DSBs (Pellicioli et al. 2001). The longer a lesion remains unrepaired, the more time for processing and production of ssDNA. Long persistence can perhaps be taken as indication of lesions difficult to repair. Third, ssDNA is generated in S phase upon stalling or nucleolytic processing of replication forks (Lopes et al. 2006; Sogo et al. 2002). All three scenarios—a high number of DNA lesions, the presence of persistent lesions and the occurence of DNA lesions or stalled/broken replication forks in S phase—can be seen as severe threat to cellular survival calling for a cell-wide response and activation of the DNA damage checkpoint. It therefore seems plausible that cells possess a counting mechanism for the ssDNA signal and that the overall amount of ssDNA in a given cell must overcome a threshold to activate the DNA damage checkpoint.

How is the ssDNA signal read and translated into a downstream DNA damage checkpoint signal? Mechanistically, ssDNA in cells is bound by RPA (Wold 1997; Chen and Wold 2014), which in budding yeast directly interacts with the DNA damage kinase Mec1 (ATR in humans) and its co-factor Ddc2 (ATRIP in humans) (Zou and Elledge 2003; Cortez et al. 2001; Paciotti et al. 2000). More ssDNA will attract more Mec1–Ddc2 (Zou and Elledge 2003; Nakada et al. 2004; Bantele et al. 2019) suggesting an intuitive mechanism of how ssDNA could be quantified. Mec1–Ddc2 phosphorylates different target proteins. Interestingly, however, not all proteins targeted by Mec1–Ddc2 show the same dependency on the ssDNA signal. In our recent work, we define two Mec1 signaling circuits, which respond differently to quantitatively different amounts of ssDNA (Bantele et al. 2019). One circuit activates the DNA damage effector kinase Rad53, which mediated by its co-sensors and scaffolds is recruited and activated in a manner that strongly depends on the ssDNA length and therefore integrates over the ssDNA signal (Bantele et al. 2019; Ira et al. 2004). As Rad53 sets off the cell-wide DNA damage checkpoint (de Oliveira et al. 2015; Harrison and Haber 2006; Branzei and Foiani 2006), we call this response the “global” signaling circuit (Fig. 1a, left). A second circuit leads to phosphorylation of the histone H2A (γH2A), which forms a chromatin domain surrounding the DNA lesion (Shroff et al. 2004; Rogakou et al. 1998) and behaves fundamentally different. γH2A phosphorylation appears to be full-blown even at very low amounts of ssDNA signal and is seemingly unresponsive to further ssDNA accumulation (Bantele et al. 2019). Notably, γH2A is not involved in the cell-wide response but rather thought to facilitate local changes in the damaged chromosome and perhaps promote repair (Downs et al. 2004; Kim et al. 2007; Kruhlak et al. 2006; Redon et al. 2003; Tsabar et al. 2015). We therefore termed this response the “local” signaling circuit (Fig. 1a, right). As both circuits are triggered by the same kinase—Mec1–Ddc2, a model emerges by which different Mec1 kinase targets have different sensitivities towards the kinase. While γH2A, in the center of the local response, is efficiently targeted already at very low levels of damage-associated Mec1 kinase, activation of the DNA damage checkpoint effector kinase Rad53, which acts globally, requires accumulation of ssDNA-RPA (Bantele et al. 2019). Such a separation of responses at different amounts of the ssDNA signal seems to be a reliable strategy to ensure immediate full-blown local repair while launching a cost-intense, cell-wide checkpoint response including cell cycle arrest only if the damage load is high or DNA lesions are persistent and a safe passage through the cell cycle is no longer guaranteed.

**Fig. 1:**
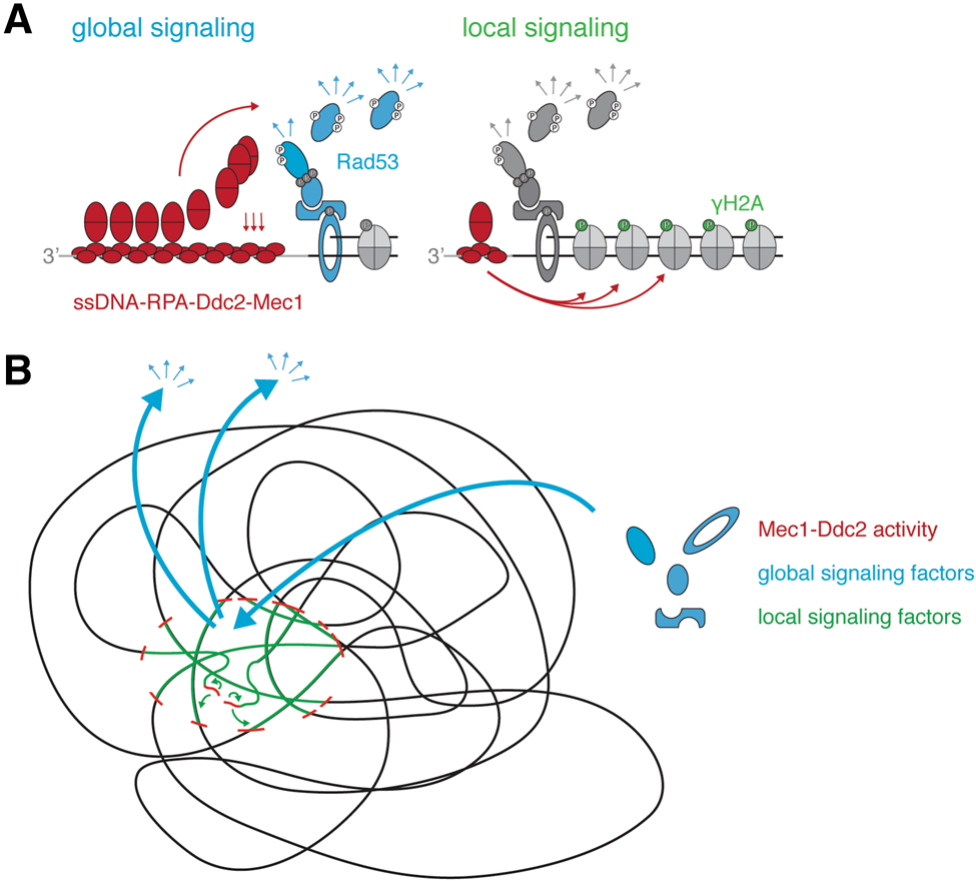
Two distinct signaling circuits operate DNA damage signaling. **a** Schematic representation of the two Mec1–Ddc2-dependent signaling circuits within the DNA damage checkpoint. The global signaling circuit (left) integrates the quantitative information of the initial ssDNA–RPA signal and over a wide range of signal translates it into a proportional amount of activated Rad53 effector kinase. The local signaling circuit (right) is already fully active at very low amounts of ssDNA–RPA signal and triggers the local response. **b** Putative model of the different mechanisms by which substrates of the local (green) and global (blue) checkpoint signaling circuits access Mec1 activity (red), which in case of local signaling is likely determined by architectural characteristics of the chromatin

The concept of signaling thresholds separating different cellular responses can be observed already in the bacterial SOS response (Michel 2005; Maslowska et al. 2019). The SOS response also monitors ssDNA accumulation and ssDNA–RecA binding and signals it via the LexA transcriptional repressor system. Here, the LexA-dependent repression of repair genes is gradually removed upon increasing RecA–ssDNA accumulation/DNA damage. At low doses of ssDNA, genes involved in nucleotide excision repair (NER) are de-repressed (Courcelle et al. 2001; Michel 2005). Further ssDNA accumulation then leads to de-repression of TLS genes and an inhibitor of cell division, which can be seen as a mechanism to stop cellular proliferation analogous to the checkpoint in eukaryotes (Tippin et al. 2004). In this case, the transition from proliferation to arrest is therefore accompanied by an additional transition from NER to mutagenic TLS (Michel 2005; Tippin et al. 2004; Courcelle et al. 2001). Interestingly, quantitative read-out of the ssDNA signal therefore seems to be a universal feature of prokaryotic and eukaryotic DNA damage responses suggesting that a decision whether DNA damage levels are tolerable or a full-blown cell-wide DNA damage response is required in all cells.

The differentiation of signaling circuits brings up several interesting questions regarding the underlying molecular mechanisms. How is Mec1 kinase activity differentially channeled towards different Mec1 targets? How is the γH2A domain established efficiently with low levels of DNA damage-associated kinase? How are thresholds that are locally defined at every lesion site integrated over spatially distinct lesion sites?

One fundamental difference between targets in the local and global circuits is their availability to phosphorylation by the Mec1 kinase. The global signaling cascade constituted by the 9-1-1 co-sensor, the Dpb11 and Rad9 scaffold proteins and the Rad53 effector kinase requires several steps of protein recruitment to the DNA lesion site, which itself is dependent on DNA end resection and Mec1 (Ira et al. 2004; Ma et al. 2006; Puddu et al. 2008). It therefore seems reasonable to suggest that protein recruitment requirements dictate subsequent phosphorylation by Mec1. In case of γH2A, the scenario is fundamentally different. H2A is an integral component of chromatin and as such immediately available as substrate for phosphorylation. Therefore, and despite the fact that histones were shown to display plasticity at DSBs (Hauer et al. 2017; Hauer and Gasser 2017; Adam et al. 2016; Tsabar et al. 2016), encounters between Mec1 and H2A over the broad γH2A domain will rather be defined by chromatin architecture and mobility than recruitment (Caron et al. 2015; Aymard and Legube 2016; Lee et al. 2013; Renkawitz et al. 2013; Zimmer and Fabre 2019). Taken together, we therefore propose that Mec1 targets are phosphorylated dependent on the frequency of kinase-substrate encounters, which in the global signaling circuit is determined by recruitment and in the local signaling circuit depends on chromatin architecture (Fig. 1b).

Based on this model, the Mec1–Ddc2 sensor module is critically involved in all ssDNA signaling circuits and likely involved in transducing the quantitative nature of the ssDNA signal. However, a second sensing mechanism might be required to confer the amount of ssDNA in the global signaling circuit. Currently, the best candidates for this second sensor are the 9-1-1 clamp and factors associated with it (Bantele et al. 2019). It has been shown that 9-1-1 is recruited to the border of resection (Majka et al. 2006; Majka and Burgers 2007), and we could observe resection-dependent 9-1-1 accumulation. However, by which molecular mechanism the 9-1-1 complex or its associated factors are involved in sensing or transducing the ssDNA signal will need to be determined by future research.

We therefore suggest that the cellular DNA damage response should not be viewed as a single pathway, but rather as separable signaling circuits. Different DNA damage scenarios trigger differential responses in these individual circuits. A quantitative understanding of the signal transduction in the individual circuits is therefore required to understand and manipulate cellular decision making upon DNA damage.
